# Positioning of Vascular Access in Pediatric Patients: An Observational Study Focusing on Adherence to Current Guidelines

**DOI:** 10.3390/jcm10122590

**Published:** 2021-06-11

**Authors:** Chiara Moreal, Rosanna I. Comoretto, Sara Buchini, Dario Gregori

**Affiliations:** 1 Unit of Biostatistics, Department of Cardiac Thoracic Vascular Sciences and Public Health, Epidemiology and Public Health, University of Padova, 35131 Padova, Italy; chiaramoreal@gmail.com (C.M.); rosanna.comoretto@unipd.it (R.I.C.); 2 EBM and EBP Sector and Professional and Organizational Development, Institute for Maternal and Child Health-IRCCS Burlo Garofolo, 34137 Trieste, Italy; sara.buchini@burlo.trieste.it

**Keywords:** vascular access, nursing, pediatric patients, adherence to guidelines, complications

## Abstract

Venous access devices (VADs) play an important role in different clinical contexts. In pediatric subjects, VAD placement is more complicated than in adults due to children’s poor cooperativity and reduced vascular access. Adherence to guidelines for the placement of VADs could prevent the occurrence of complications, but data in the literature are general and not exhaustive, especially with regard to the pediatric population. The objective of this study was to assess adherence to guidelines for the placement of VADs in a pediatric setting. A retrospective observational study was conducted in the general ward of a pediatric hospital in the northern region of Italy. Data related to consecutive admissions in the period from 1 January to 31 December 2019 were collected according to the availability of clinical documentation. A cohort of 251 subjects was considered, yielding a total of 367 VADs. Device permanence in situ and the effective administration of intravenous therapy were associated with an increased risk of complications, while adherence to guidelines was an important protective factor. Adherence to guidelines for the placement of VADs is an independent and positive predictive factor for the prevention of complications due to the presence of a vascular device.

## 1. Introduction

Vascular access devices (VADs) are among the most discussed topics in healthcare services because of their important role across all disciplines in children and adults in both inpatient and outpatient settings. The positioning of vascular devices, in various forms, represents the most common invasive procedure performed in secondary care [1], such as specialist settings.

In particular, children require vascular access for many indications, including hydration, infusion of parenteral nutrition, administration of medications, and obtaining blood samples for laboratory analysis [2]. However, it is important to consider that phlebotomy in children is an invasive and stressful procedure, usually causing fear, anxiety, and insecurity [3].

Risks associated with intravenous devices can vary depending on which device is selected. Even after choosing the lowest-risk device, such as peripheral VADs, patients can suffer from multiple attempts at insertion and re-sites, all of which can be avoided if critical thinking is applied early in selecting the most appropriate device for treatment [4]. Furthermore, assessing the right device in elective conditions in pediatrics is suggested by the most recent guidelines to facilitate the choice of the least invasive procedure based on the specific treatment prescribed and its duration [5].

Peripheral intravenous cannula is the most commonly used device in hospitalized patients [6]. The correct criteria for appropriate peripheral VAD are short-term preventive use (less than five days) and administration of nonirritant infusion therapies [7]; inadvertent administration of irritant or vesicant substances into a peripheral vein can result in tissue-damaging necrosis, requiring surgical intervention to treat [8]. The condition of difficult intravenous access (DIVA) is widespread among both adults and children [9] and is defined as a clinical situation where multiple attempts or special interventions are required to obtain and maintain peripheral venous access [10,11]. A systematic review suggested that the DIVA score should be implemented in pediatric settings to prospectively identify children at risk for difficult vascular access [12].

The characteristics of infusion therapy, such as low pH and high osmolarity, are recognized as risk factors for peripheral vein infusion-related phlebitis [13].

Spina et al. proposed, in the Italian context, high- and intermediate-risk drug lists produced by the Infusion Nursing Society (INS) Task Force on cytotoxic substances that can cause tissue damage [14]; the complete list is reported in Table S1.

In a recent review, an evidence-based index of low-risk noncytotoxic vesicant medications and solutions was developed, expanding the previous list cited in a second list, reported in Table S2 [15].

Central venous catheters are used to deliver intravenous medications, are essential for diagnostic and therapeutic interventions and are indicated in patients with limited or difficult peripheral access, including those requiring frequent blood collection [16]. Several associations have defined appropriateness criteria and guidelines in VAD management [7,17,18,19,20]. Baskin et al. [21] suggested a multidisciplinary approach to venous access care in patients with chronic VAD insertion. In their recommendation, venous access planning should begin at first diagnosis, defining the need for acute or chronic use (Grade C, Class IIa).

VADs are identified as the most common device associated with hospital admission for adverse medical events in pediatrics, both peripheral and central [22]; the most frequent catheter-related complications (in particular, for central VADs) are bloodstream infection, dislodgement, occlusion, thrombosis and insertion site infection [23]. A significant association between inappropriate device placement and an increased risk of adverse device-related complications has already been described [24], and recommendations to remove catheters promptly when no longer deemed clinically necessary are strong [25].

Adherence to guidelines for the placement of VADs could prevent complications, premature device failure, vessel damage and venous depletion, but to the best of our knowledge, there is an important lack of information about this topic, especially in the pediatric context.

The objective of this study is to evaluate the association between adherence to current guidelines for VAD placement and the development of complications in hospitalized children.

## 2. Methods

### 2.1. Setting and Population

This observational retrospective study was conducted in the Friuli—Venezia Giulia Region, northeastern Italy. Data were collected from consecutive medical charts (according to the availability list provided by the structure) of subjects hospitalized in the general pediatric ward of Burlo Garofalo Pediatric Hospital in Trieste.

Data were collected anonymously on an electronic case report form by means of the web software REDCap (Research Electronic Data Capture) in collaboration with the Unit of Biostatistics, Epidemiology and Public Health, Department of Cardio-Thoracic-Vascular Science and Public Health of Padua University.

The study did not involve direct patient contact. Informed consensus through the GE.CO. system (in use in the region considered) for the use of clinical data for purposes of clinical research had to be obtained before conducting the present study, which was required and considered valid.

The inclusion criteria were age between 29 days and 16 years at the time of last admission, admission to the selected care unit between 1 January 2019 and 31 December 2019, and the presence of signed informed consent to the treatment of data for scientific reasons through the GE.CO. system given by legal guardians of the subjects. Patients were excluded from the study in case of absence of one of the inclusion criteria, subjects in replacement renal therapy and arterial devices and/or arterial blood sampling.

The variables collected can be divided into three groups: demographics and anthropometric characteristics (date of birth, sex, weight, and height); disease and therapy characteristics (primary disease, diagnosis of admission and of discharge, date of admission and discharge, timing and drugs of intravenous therapy, venous blood sampling collected, qualities of vascular patrimony subject); and VAD characteristics (presence/absence, type, lifetime, complications). Data on all types of intravenous (IV) therapy, including those according to lists of injectable substances recognized as dangerous for peripheral veins, were also collected. These drugs are presented in Supplementary Tables S1 and S2, called “List A” and “List B,” respectively.

The criteria used for defining adherence to clinical guidelines were (i) drug infusion in central VADs for drugs associated with endothelium damage [6]; (ii) intravenous therapy length over six days in central VADs [7]; and (iii) removal of a VAD when not required [26].

### 2.2. Statistical Analysis

A basic descriptive data analysis is reported using the median (I–III quartile) for continuous variables and the percentages (absolute numbers) for categorical variables, as appropriate. Unadjusted differences were tested using Wilcoxon or chi-square tests without continuity corrections whenever appropriate, depending on the variable analyzed.

The effects of relevant confounders on complications were considered by estimating a multivariable longitudinal linear model. Variables were selected from a pool of significant variables by univariable analysis (at least 0.25) using the Akaike information criterion in a forward fashion and a threshold for significance of 0.10 [27]. Nonlinear effects of covariates were estimated using restricted cubic splines, and their significance was estimated using a log-likelihood ratio test. Goodness of fit was evaluated using Somer’s *D* and *R*^2^ on a set of bootstrapped (*B* = 10,000) resamples. The analysis was performed using the RMS libraries [28] and the R System [29].

## 3. Results

The study sample included 251 subjects, yielding a total of 367 admission events observed (from 1 to a maximum of 5 events per subject). Among all admissions, 270 VADs were explored (flowchart reported in Figure S1). Table 1 reports the characteristics of subjects and their distribution. Table 2 describes admission events stratified by guideline adherence behavior when using VADs.

A significant difference between the two groups (nonadherent vs. adherent to guidelines) was observed in length of stay (*p* < 0.001), duration of intravenous therapies (*p* < 0.001), presence of venous access (*p* < 0.001), and complications (*p* = 0.005). In eight occurrences, it was not possible to evaluate adherence to guidelines because no data concerning VAD were retrieved.

In Table 3, the characteristics of VADs, according to the development of complications during hospitalization, are reported. Differences between the two groups were observed in length of stay (*p* < 0.001), infusion of intravenous therapies (*p* < 0.001), and adherence to guidelines (*p* = 0.003).

**Figure 1:**
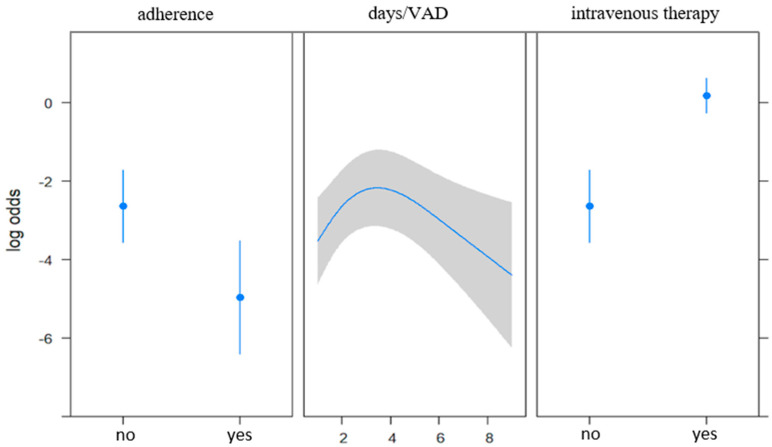
Association between the occurrence of complications and adherence, days/VAD and administration of intravenous therapy. Nonlinearity for days/VAD (*p* = 0.0014) was estimated via restricted cubic splines and was adjusted for adherence and administration of intravenous therapy. Abbreviation: VAD, vascular access device.

A multivariable model was implemented to estimate the association between the presence of complications and guideline adherence, VAD dwelling days, and administration of intravenous therapy (Table 4 and Figure 1).

A significant negative association was observed between the presence of complications and adherence to guidelines (*p* < 0.0001), while for the other two factors, a positive association was observed (*p* < 0.0001 and *p* = 0.006 for the administration of intravenous therapy and VAD dwelling days, respectively). The effect of VAD dwelling days was still significantly nonlinear (*p* = 0.0014).

## 4. Discussion

Decisions about vascular access placement were found to be a relevant topic associated with the occurrence of complications in pediatric inpatients. Although adherence to guidelines for the placement of VADs could prevent several complications and vessel damage [23], to the best of our knowledge, no studies regarding this topic have been performed, especially in the pediatric context.

Therefore, this study focused on adherence to guidelines for VAD placement, which include evaluation of the appropriateness of devices to be inserted, depending on the clinical needs and characteristics of both patients and infusion therapies. It is important now to underline that all placement procedures (in particular, for central VADs) were performed in accordance with the hospital’s institutional policy following practical guidelines on this topic (i.e., operating room setting, anesthesiology assistance for sedation, use of personal protective equipment, skin antisepsis, ecographic technique, and sutureless device fixation).

In this study, nonadherent cases seem to be associated with a prolonged length of stay, which can increase several risks related to the hospital setting, costs for the healthcare system, and the psychological suffering of children [3,21]. However, a prolonged duration of hospitalization may also be affected by other factors not considered in this analysis, such as the severity of the disease. In fact, the decision to use intravenous therapy is more often based on the severity of the disease, which likely leads to a longer hospital stay alone.

The first aspect used to define nonadherent cases was represented by therapies prolonged over 6 days. In the observed institute, no protocols or procedures were found that regulate in detail the replacement of vascular access; in addition, it should be considered that some devices may have been removed and repositioned, but these actions might not have been documented. Although the gold standard is represented by the less invasive procedure, in this case, a central VAD, or alternatively, *midline* in case of nondamaging substances, is required [16,29]. Advanced technology, such as the use of ultrasound and intracavitary electrocardiography, has made these procedures safer and more comfortable for patients. However, it is important to emphasize that the placement of both a central VAD and peripherals, such as the midline, requires advanced personnel skills and organizational resources that are not always present in all hospital settings.

However, data from this study revealed that peripheral devices were often inappropriately present. Furthermore, more infusions of drugs belonging to list B have been shown to be administered in the nonadherence group than in the adherence group. Similarly, a significantly higher number of complications (in particular, phlebitis) occurred in the nonadherence group. However, other factors, such as the disease severity and the activity level of the patient, should be considered in the evaluation of complications. No differences were observed in VAD dwelling days with respect to adherence patterns.

Intravenous therapy is recognized as an important variable related to the occurrence of complications: obviously, the infusion of substances through all devices, especially through peripheral devices, can increase adverse phenomena, such as phlebitis [30]. The results from this study are in accordance with data previously reported, as a significantly higher percentage of irritant and vesicant substances, such as those that were included in lists A and B, was found in the cases where complications were recorded. Furthermore, the multivariable model revealed a significantly increased risk for complications if intravenous infusions were administered. Similarly, the length of stay was significantly longer, and the administration of drugs included in both lists A and B was observed more frequently in case of complications. In particular, greater than 60% of complications were recorded when the drugs included in list B, which are not yet considered in the current guidelines, were administered. These results support those reported by Gorski [31] regarding the importance of the administration of these drugs through a central VAD to avoid serious complications. Furthermore, these data reflected an important scenario in which the majority of drugs in list A are usually administered in intensive care units, whereas those in list B are more often administered in general wards. Consequently, we could expect higher frequencies of peripheral VADs (and therefore a higher rate of drugs in list B) in non-ICU settings, as we observed in the unit explored.

Observing the results of the multivariable model, adherence can be considered a strong and independent factor correlated with the occurrence of complications in the pediatric population. This association was already reported by Tiwari, who found a strong link between the inappropriate use of devices and adverse device-related outcomes [32]. Furthermore, a negative association has been observed from our data, in line with the guidelines’ recommendations, indicating that adherent behavior could be a protective factor against complications. In clinical practice, this can translate into fewer feelings of discomfort and a better quality of life for children, as well as reduced resource consumption for the healthcare system.

Similarly, the dwelling catheter’s time has been demonstrated to be a predictor of complications in a nonlinear relationship, with maximum occurrences on the third day; the subsequent slope is likely linked with the few observations after that time point, mainly observed in peripheral VADs. Guidelines suggest the prompt removal of VADs when not necessary, as longer catheterization is associated with the development of complications [24]. Lastly, a high number of devices were not associated with the administration of injectable drugs. Nonuse of these devices could be explained by the clinical practice of healthcare personnel to obtain blood samples from catheters to avoid venipuncture, especially in the pediatric population, even if this practice can lead to the collection of hemolyzed specimens [33]. In fact, routine blood sampling is described as an inappropriate use of peripheral VADs [8]. Nevertheless, to the best of our knowledge, no consensus exists concerning the rates of hemolyzed specimens obtained using peripheral VADs.

### Strengths and Limitations

This is the first study investigating the relationship between adherence to guidelines for VAD positioning and the development of complications in pediatrics. Furthermore, this is the first study that sought to explore these issues in the Italian setting, and therefore, the results can act as a starting point for future research regarding the use of VADs and the development of specific training projects aimed at healthcare personnel.

This study also has several limitations. First, subjects and related hospitalization records were not randomized. However, analysis was performed to account for potential confounding factors using a multivariable model, and the selection procedure, although not randomized, was entirely based on a consecutive list of patients. Second, data were collected from a single general care unit of a pediatric hospital (and they did not include sick neonates, premature infants, or critically ill children). Therefore, these results cannot be generalized to other care units or to a national level. Finally, it seems important to point out that no information could be retrieved regarding the characteristics of the vascular access of patients or about the number of attempts for success in the positioning of peripheral VAD. These outcomes were reported as factors that may influence the requirement of a venous line, suggesting the use of advanced techniques, such as ultrasound, to obtain it [34]. Further studies should be conducted to explore this topic and investigate its role.

## 5. Conclusions

This study revealed that adherence to VAD placement guidelines, which represents the gold standard in quality of care, should be recognized as an independent and predictive factor for the occurrence of complications in hospitalized pediatric subjects.

Therefore, it is important that healthcare personnel, particularly nurses who are responsible for drug administration, constantly monitor aspects related to the patient’s vascular access and the actual need for a specific type of vascular access. The right and early choice of the appropriate device based on all of these factors can lead to a decreased number of complications and, therefore, to a better quality of life for hospitalized children.

## Figures and Tables

**Table 1:** Subject characteristics. Data are presented as the median (I, III quartile) for continuous variables and as percentages (absolute numbers) for categorical data.

Variables	*n* = 251
**Gender (Male)**	48.2% (121)
**Age (y)**	7 [2;13]
**Height (cm)**	123 [98;152]
**Weight (kg)**	23 [12.8;42]

**Table 2:** Total admission characteristics according to adherence to guidelines for the placement of VADs. Data are presented as the median (I, III quartile) for continuous variables and as percentages (absolute numbers) for categorical data.

Variables	Nonadherence (*n* = 207)	Adherence (*n* = 152)	Combined ^§^ (*n* = 359)	*p*-Value
**Length of stay (days)**	3 [2;7]	2 [1;3]	3 [2;5]	<0.001
**IV therapy (Yes)**	53% (110)	43% (65)	48% (175)	0.067
**IV therapy > 6 days**	45% (50)	12% (8)	33% (58)	<0.001
**List A infusion**	8% (16)	5% (8)	7% (24)	0.355
**List B infusion**	38% (75)	3% (4)	23% (79)	<0.001
**VAD presence (Yes)**	98% (202)	50% (76)	77% (278)	<0.001
**VAD type**				0.205
**Central**	3% (5)	4% (5)	4% (10)	
**Peripheral**	98% (195)	86% (69)	95% (264)	
**NA**	1% (2)	2% (2)	1% (4)	
**Days/VAD**	2 [1.25;4]	2 [2;3]	2 [2;3]	0.604
**Complications (all)**	29% (56)	12% (9)	24% (65)	0.005
**Accidental removal**	6% (12)	1% (1)	5% (13)	0.117
**Phlebitis**	22% (44)	6% (5)	18% (49)	0.003

^§^ The overall 367 admission events were reduced to 359 because in 8 cases, no data concerning VAD were retrieved.

**Table 3:** Characteristics of the overall events (i.e., number of VADs) according to the presence of complications that occurred during hospitalization. Data are presented as the median (I, III quartile) for continuous variables and as percentages (absolute numbers) for categorical data.

Variables	Complications (*n* = 65)	No Complications (*n* = 205)	Combined (*n* = 270)	*p*-Value
**Admission days**	5 [3;7]	2 [3;5]	3 [2;5]	<0.001
**IV therapy**				<0.001
**No**	8% (6)	46% (94)	37% (100)	
**Yes**	92% (59)	54% (111)	63% (170)	
**List A infusion**	15% (10)	6% (13)	8% (23)	0.023
**List B infusion**	63% (41)	19% (37)	30% (78)	<0.001
**VAD presence**				0.706
**Yes**	98% (64)	99% (203)	99% (267)	
**No**	2% (1)	1% (2)	1% (3)	
**NA**	0% (0)	0% (0)	0% (0)	
**Days/VAD**	3 [2;4]	2 [1;3]	3 [2;3]	0.052
**VAD type**				0.812
**Central**	3% (2)	4% (8)	4% (10)	
**Peripheral**	97% (63)	96% (196)	96% (259)	
**NA**	0% (0)	0% (1)	0% (1)	
**Adherence (Yes)**	14% (9)	33% (67)	28% (76)	0.003

Abbreviations: IV, intravenous; VAD, vascular access device; NA, not available.

**Table 4:** Multivariable model for the occurrence of complications.

	Odds Ratio	Lower 0.95	Upper 0.95
**Adherence (Yes vs. No)**	0.098	0.037	0.256
**Days/VAD (1 point difference)**	1.531	1.107	2.118
**IV therapy (Yes vs. No)**	16.795	6.002	47.001

Effect is the slope of the linear regression model for each covariate. The effect is expressed in terms of the interquartile difference for days/VAD covariate and using a reference category for categorical variables (for days/VAD, the *p*-value for nonlinearity was 0.0014). Abbreviations: IV, intravenous; VAD, vascular access device.

## Data Availability

The data presented in this study are available upon request from the corresponding author.

## Supplementary Materials

The following are available online at https://www.mdpi.com/article/10.3390/jcm10122590/s1: Figure S1: Flowchart representing the distribution of events; Table S1: List A: high- and intermediate-risk lists produced by the Infusion Nursing Society Task Force on the cytotoxic substances of cancer therapies that can cause tissue damage as reported by Spina et al. [14]; Table S2: List B: noncytotoxic infusates listed in systematic reviews as vesicants not retained on the Infusion Nursing Standards Vesicant list, traced by Gorski et al. [15].
