# Supplementary material for: Positioning of Vascular Access in Pediatric Patients: An Observational Study Focusing on Adherence to Current Guidelines
Source: J Clin Med. 2021 Jun 11;10(12):2590. doi: 10.3390/jcm10122590 (PMC8230876; doi:10.3390/jcm10122590)
Supplement: Supplementary file 1 [file jcm-10-02590-s001.zip › Figure S1.pdf]

Figure S1. Flow chart representing the distribution of events.

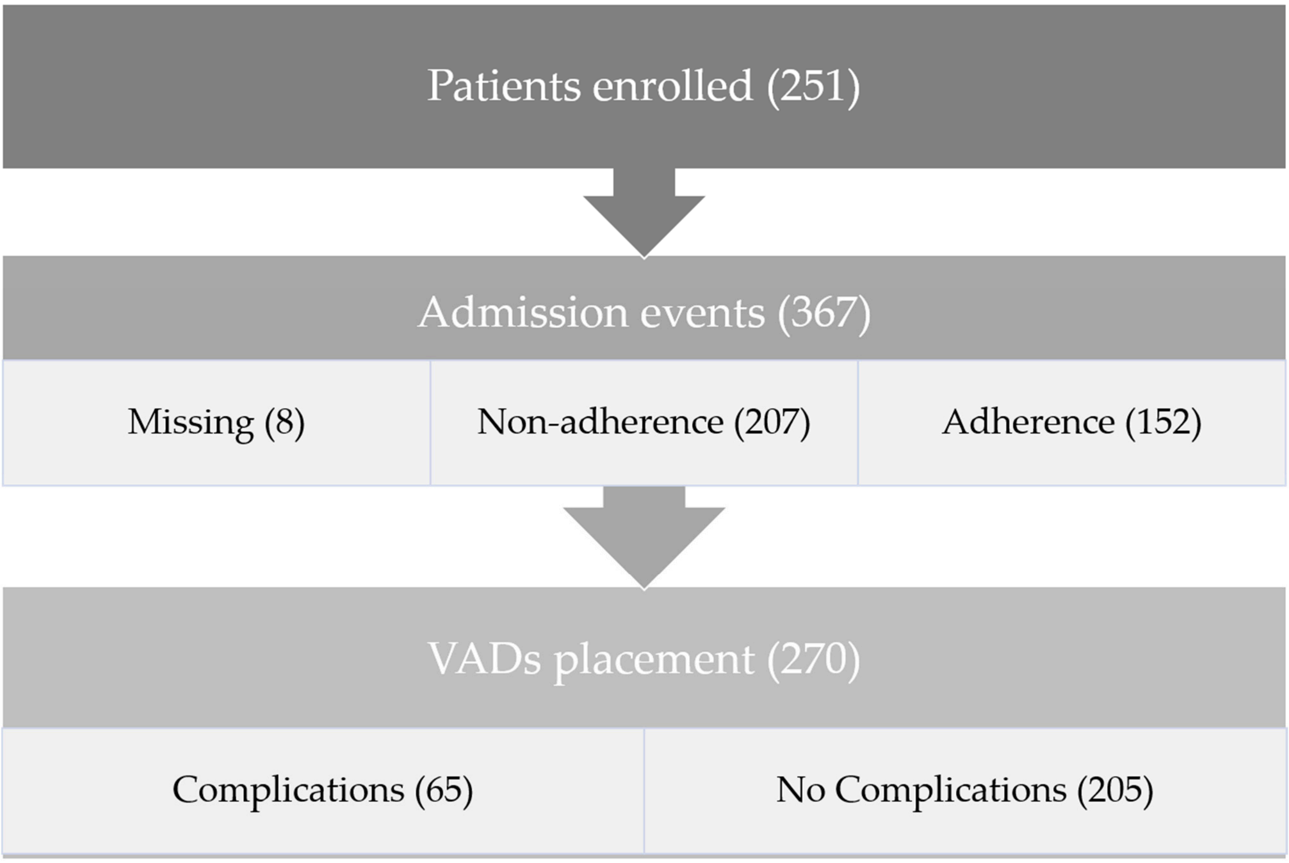

Abbreviations: VADs, vascular access devices.
