# Supplementary material for: Positioning of Vascular Access in Pediatric Patients: An Observational Study Focusing on Adherence to Current Guidelines
Source: J Clin Med. 2021 Jun 11;10(12):2590. doi: 10.3390/jcm10122590 (PMC8230876; doi:10.3390/jcm10122590)
Supplement: Supplementary file 1 [file jcm-10-02590-s001.zip › Table S1.pdf]

Table S1

List A: high and intermediate risk lists produced by the Infusion Nursing Society Task Force on the cytotoxic substances of cancer therapies that can cause tissue damage as reported by Spina et al. [14].

High risk<br>**Drugs clearly recognized to be vesicant, with numerous references in the literature and numerous reports of tissue damage**	*Intermediate risk*<br>**Vesicant medications associated with fever extravasation reports published; information on the drug and its characteristics indicate caution for potential tissue damage**
• Calcium chloride<br>• Calcium gluconate<br>• Non-ionic contrast media<br>• Dextrose in ≥ 12.5% concentration<br>• Dobutamine<br>• Dopamine<br>• Epinephrine<br>• Norepinephrine<br>• Solutions for parenteral nutrition higher than 900 mOsm/L<br>• Phenylephrine<br>• Phenytoin<br>• Prometazione<br>• Sodium bicarbonate<br>• Sodium chloride ≥ 3%<br>• Vasopressin	• Acyclovir<br>• Amiodarone<br>• Arginine<br>• Dextrose in a concentration of ≥ 10% but < 12.5%<br>• Mannitol ≥ 20%<br>• Nafcillin<br>• Pentamidine<br>• Sodium pentobarbital<br>• Sodium phenobarbital<br>• Potassium ≥ 60 mEq/L<br>• Vancomycin hydrochloride
