# Supplementary material for: Positioning of Vascular Access in Pediatric Patients: An Observational Study Focusing on Adherence to Current Guidelines
Source: J Clin Med. 2021 Jun 11;10(12):2590. doi: 10.3390/jcm10122590 (PMC8230876; doi:10.3390/jcm10122590)
Supplement: Supplementary file 1 [file jcm-10-02590-s001.zip › Table S2.pdf]

Table S2

List B: Non-cytotoxic Infusates Listed in Systematic Reviews as vescicants not retained on Infusion Nursing Standards Vescicant list, tract by Gorski at al. [15].

**Infusated**	Listed in Le and Patel (2014)	Listed in Clarck et al. (2013)	Listed in Reynolds et al. (2014)	Rationale for not Retaining on vescicant list
Albumin	Yes; 1 citation	No	No	Single citation where researchers stated that some medications and fluids were being co-infused through the same line when the extravasation injury occurred which made it difficult to determine the offending agent
Aminophylline	No	Yes, 1 neonatal citation, rated as lower risk	Yes; Single 1981 citation; unable to retrieve	Limited citations; rated as lower risk
Amphotericin	Yes	Yes, intermediate risk; no evidence of permanent harm in pediatric literature	No	Limited citations; mechanism of tissue damage in a single adult report suggested cause as mechanical compression due to increase fluid
Ampicillin	Yes; 1 citation	Yes, lower risk; evidence of harm in pediatric literature	Yes; Single 1981 citation; unable to retrieve	Limited citations; rated as lower risk
Cloxacillin	Yes; 1 citation	No	No	Single citation where researchers stated that some medications and fluids were being co-infused through the same line when the extravasation injury occurred which made it difficult to

				determine the offending agent
Doxycicline (tetracycline)	Yes; 2 citations	Yes (higher risk); No references; local consensus	Yes; Single pediatric citation	Limited citations
Furosemide	Limited citations; rated as lower risk	Yes, neonatal citation rated as lower risk	No	Limited citations; rated as lower risk
Gentamicin	Limited citations; rated as lower risk	Yes, lower risk; evidence of harm in pediatric literature	No	Limited citations; rated as lower risk
Immunoglobulin	Limited citations; rated as lower risk	Yes, 1 citation (lower risk, evidence of harm in pediatric literature)	No	Limited citations; rated as lower risk
Lorazepam	Yes; 1 citation	Yes, pediatric citation rated ad intermediate risk, no evidence of permanent harm	Yes, no citation to support	Two citations; no evidence of permanent harm in pediatric literature
Metronidazole	Yes, single citation; very large extravasation into dorsum of hand (50 mL)	No	No	Single citation
Morphine	Yes; 1 citation	Yes, neonatal citation rated as intermediate risk	No	Limited citation; no evidence of permanent harm in pediatric literature; morphine is routinely administered subcutaneously in chronic/palliative pain management; Single citation
Oxacillin	Yes; 1 citation	No	No	Single citation

Penicillin	Yes; 1 citation	No	No	Single citation
Propofol	Yes, several citations	No	No	Limited citations
Sodium thiopental	Yes, two audit citations	No	Yes; single pediatric citation	Limited citations
Sodium valproate	Yes; 1 citation	No	No	Single citation
